# Intergenerational status transfer and post-compulsory pathways in a changing education system: a comparison of two Swiss school-leavers' cohorts

**DOI:** 10.3389/fsoc.2025.1585464

**Published:** 2025-09-01

**Authors:** Andrés Gomensoro, Sandra Hupka-Brunner, Thomas Meyer

**Affiliations:** ^1^TRansitions from Education to Employment (TREE), Institute of Sociology, Department of Social Sciences, University of Bern, Bern, Switzerland; ^2^Institute of Demography and Socioeconomy (IDESO), Geneva School of Social Sciences, University of Geneva, Geneva, Switzerland

**Keywords:** occupational status, intergenerational social mobility, vocational education and training, Switzerland, cohort data

## Abstract

**Introduction:**

This study compares the mechanisms of intergenerational occupational status transfer among a subsample of two Swiss school-leaving cohorts (2000 and 2016) that was enrolled in VET programmes at upper-secondary level of education. In particular, we focus on the effect of tracking at the lower-secondary level, which is pronounced in Switzerland and known to play a crucial role for educational and social stratification.

**Methods:**

Drawing on data from two cohorts of the TREE^1^ panel survey, we compare cohort-specific linear regression models that examine overall intergenerational transmission of status and direct as well as indirect or mediating effects of lower-secondary track attendance on (occupational) status attainment. As a measure for analyzing the association between parental and child status, we draw on the recently developed linear hierarchical Occupational Earnings Potential (OEP) scale.

**Results:**

We find that while the impact of lower-secondary tracking has declined across cohorts, overall intergenerational status transmission remains stable, suggesting that institutional context factors that have changed across cohorts (such as educational reforms and improvements of VET supply) have failed to mitigate intergenerational status transmission and social inequality.

**Discussion:**

Our analyses underscore the need for structural policy interventions to enhance educational equity, particularly measures that foster de facto (and not only formal) permeability within the highly stratified Swiss education system.

## 1 Introduction

Most societies are characterized by some form of social stratification, which can be defined as a hierarchy between the members of a society that leads to an unequal distribution of resources and life opportunities. This stratification is deeply rooted in the division of labor and reflected through the specifics of occupations associated with different levels of status and prestige (Oesch et al., 2025). According to the O-E-D^2^ triangle originally put forward by (Blau and Duncan 1967), education is a key factor for status attainment, but in its turn significantly influenced by the “O” ([social] Origin). It is widely held that no society succeeds in eliminating the influence of social background on the educational success of children. However, there are significant cross-national differences with regard to the extent of this influence (OECD, 2018a,b), which in turn raises the question of what role educational systems play in the reproduction of social inequalities. In order to understand status attainment, social mobility and inequality, we also need to take into account the mechanisms and regimes at work throughout individuals' transitions within the education system (transitions between levels and/or programmes) and from education to the labor market.

Furthermore, both education and transition systems in modern knowledge-based societies and economies have undergone fundamental changes in the past decades. One of the most significant changes is educational expansion, “an enhancement and an increase in the size of educational systems, an increase in educational opportunities and a rising demand for education,” as (Hadjar 2019) puts it. According to (Hadjar and Gross 2016; see also Bukodi and Goldthorpe, 2018), educational expansion ought to be linked to an increased equity of educational opportunities and hence a decreased influence of social origin on life opportunities. If this is true, we should expect a decreasing impact of social origin on educational attainment between different age cohorts.

Nevertheless, (van de Werfhorst 2024) points out that not all social classes benefit equally from educational expansion. Moreover, class-specific differences not only persist with regard to educational achievement, but also in terms of returns on education (Bukodi and Goldthorpe, 2018; van de Werfhorst, 2024). In the course of educational expansion, educational systems often have aimed at implementing reforms to decrease social inequalities, but also to adapt to new societal needs and challenges (Hadjar and Gross, 2016). Whether and to what extent these educational policy efforts and changing societal macro-contexts have contributed to a reduction of social inequalities is open to debate.

Against this background, this paper investigates the transition from initial formal education into the labor market of two Swiss age cohorts who have left compulsory education in 2000 and 2016 respectively. Comparing the two cohorts whose trajectories lie 16 years apart allows us to account for the macro-context in which educational pathways and transitions are embedded. In Switzerland, this context has witnessed substantial changes, including reforms of the education system which can be expected to affect youths' educational trajectories (see Section 3.3). We draw on data from the Swiss panel survey TREE (Transitions from Education to Employment), adopting a new scale that captures the earning potential of occupations (OEP). We examine changes in the extent to which social origin influences educational attainment and hence successful transition to the labor market. In doing so, we particularly focus on the effect of tracking at lower-secondary level of education, which is pronounced in Switzerland and known to play a crucial role for social stratification (see Section 3 for more detail).

## 2 Theoretical background and state of research

In the present scientific discourse, there is a lively debate on how social reproduction and the role of education with regard to social mobility has evolved and changed over the past decades. Drawing on Blau and Duncan's paradigm of the O-E-D triangle, various scholars are discussing whether social mobility patterns have changed over time, and to what degree (formal) education and particularly educational expansion has contributed to this change (Bukodi and Goldthorpe, 2018, 2024; van de Werfhorst, 2024). The respective findings and assessments are inconclusive. Drawing on ESS^3^ data from 40 countries, (van de Werfhorst 2024) holds that, owing to the educational expansion of the past decades (birth cohorts 1920–1979), intergenerational transmission of (occupational) status has indeed weakened. Relying on a comparative analysis of 17 European countries, (Bukodi and Goldthorpe 2024) challenge this assertion. According to them, the weakening of intergenerational status transmission is by and large restricted to the age cohorts of the “*trente glorieuses*,” i.e., the period between the end of World War II and the mid-1970s. Theoretically, they hold that the O-E-D triangle cannot be properly assessed without taking into account changes in the structure of class and of the labor market. Against this background, (Bukodi and Goldthorpe 2018, p. 15) claim that the weakening of the O-D association is likely to be due to the decline of the agricultural sector during the “*trente glorieuses*,” which is known for its strong propensity for intergenerational immobility.

As to the role that education and its institutional settings plays in the mechanisms of the O-E-D triangle, (van de Werfhorst 2024) identifies, beyond educational expansion, two factors that have an equalizing effect on intergenerational status transmission: a higher school-leaving age and a later tracking age. He further holds that he finds no evidence for what he calls the “direct persistence” theory, i.e., that privileged families always find a way to counteract institutional measures aiming at the reduction of social inequality (with particular respect to tracking at secondary school levels, see also: Triventi et al., 2016, 2020). (Triventi et al. 2020), who draw on comparative findings across 17 countries, conclude that socio-economically advantaged families tend to successfully secure educational “pole positions” for their children across different types of school systems. In a similar vein, (Bukodi and Goldthorpe 2024) stress the importance of the capacity of more advantaged parents to protect their children against (potential) downward mobility.

Evidence regarding Switzerland is scarce, patchy and (also) inconclusive. Drawing on an analysis of eight different birth cohorts, (Falcon 2013) finds no evidence for any increase of intergenerational social mobility over time and concludes that unlike other industrialized countries, inequality based on social origin remains persistent in Switzerland. Föllmi and Martìnez (2017) hold that due to low educational mobility by international standards, intergenerational income mobility in Switzerland is lower than in most other European countries.

(van de Werfhorst 2024) study that has already been cited above also includes data from Switzerland. It compares birth age cohorts from 1920 to 1979 with regard to overall intergenerational status transmission on the one hand and the share of “indirect” transmission via education on the other. For the case of Switzerland and in line with his general findings across all countries under study, he finds a notable decrease of overall transmission and a slight increase of the “indirect path” for the cohorts born before 1960 (van de Werfhorst, 2024, p. 17). Contrariwise, overall transmission in Switzerland is on the rise again for the cohorts born in the 1960s, while the level of the “indirect path” remains stable. For the cohorts born in the early 1970s, the level of overall transmission is again declining, while the effect of the “indirect” path is on the rise.

As to the impact of parental status on education, (Hadjar 2019) has conducted a study including German and Swiss cohorts born between 1925 and 1974, in which he finds that comparatively, educational opportunities have improved the most substantially among the Swiss working class. Recent findings, however, suggest that with respect to student achievement, the impact of social origin, remains persistent and strong by international standards. Recently published results from PIAAC,^4^ the OECD's large scale assessment of literacy skills among the adult population, reveal that along with Germany, Switzerland ranks at the top of participating countries with regard to the discrepancy between scores of respondents whose parents have attained a high vs. low level of education (OECD, 2024a). PISA results on their part show that among the 15-years-old, the social origin gap of Swiss performance scores, already markedly above average in international comparison, has even widened in the past decade (Erzinger, 2023; OECD, 2023). In a cross-cantonal comparison, (Schmutz 2024) finds that the level of (social) inequality correlates with institutional features such as secondary school stratification. According to her, higher stratification of a cantonal school system is associated with greater inequality of opportunity (without improving overall student achievement). Even though these recent studies on the impact of social origin on student achievement do not allow for any conclusions on the “final” (status) destination, they are valuable as intermediate indicators of intergenerational status transmission. This is particularly true for the case of the Swiss education/transition system with its marked path dependencies which will be outlined in Section 3.

Most of the presented findings have two major shortcomings in common: Firstly, they are “historical” and thus reflect O-E-D mechanisms that lie largely in the past. Secondly, they mostly rely on cross-sectional and/or retrospective data, leaving us with the flaw that most of the intermediate steps and processes between “origin” and “destination” remain a black box. In the case of Switzerland, the TREE data hold the potential to remedy both of these shortcomings: following up on cohorts that have left compulsory school in 2000 (1st cohort) and 2016 (2nd cohort) respectively, they reflect intergenerational (im)mobility processes that lie in a very recent past (in the case of the 1st cohort) or are still ongoing (2nd cohort). Furthermore, the cohorts' seamless longitudinal observation allows us to account for critical intermediate steps on the way between “origin” and “destination,” such as transitions not only from school to work, but also within the trajectories of formal education.

The theoretical framework of the TREE study and hence the TREE data (Hupka-Brunner et al., 2022) essentially draws on (Bernardi et al. 2019)'s life course paradigm, which accounts for the fact that the effects of known mechanisms (in our case: the role of education for social reproduction) may assume different shapes if their social context changes over time. With their multi-dimensional life course cube,^5^ Bernardi et al. provide a highly valuable conceptual scheme that has made a major contribution to creating a uniform terminology and structuring the theoretical debate. Bernardi et al.'s paradigm highlights the embeddedness of individual development in historical and local contexts that strongly affect opportunity structures and institutional frameworks in which individuals must act and decide.

## 3 The Swiss context now and then

In order to gain an adequate understanding of intergenerational status transmission in Switzerland and thus the mechanisms at work within the Swiss O-E-D triangle, we need to have a solid notion of the main specifics of its societal macro-context. In the following, we therefore present a brief overview of the Swiss education system, the Swiss labor market and the transition system between the two. As we analyse two cohorts having left compulsory school 16 years apart (2000 and 2016), we also account for major changes of the macro-context throughout the period in question.

### 3.1 Education system

The Swiss education system is characterized by a federal, small-knit structure, a pronounced horizontal and vertical stratification from lower-secondary level onwards, the dominance of dual, market-based VET, and a relatively low participation rate at tertiary level, especially in the vocational education and training (VET) sector.

Compulsory education in Switzerland comprises 6 years of primary and 3 years of lower-secondary education.^6^ A first important setting of the course takes place at around 12 years of age, when students make the transition from primary to lower-secondary school: In most cantons, students are divided into two to four different tracks of lower-secondary school, which differ according to their curricula and level of academic requirements. The extent and form of tracking varies greatly from canton to canton. Approximately 30 per cent of all students are assigned to tracks of the so-called ‘basic requirements' type, with inter-cantonal variation ranging from 10 to 40 percent.^7^ Even though educational policy postulates permeability between tracks, the initial allocation to a given track remains largely irreversible (Bayard, 2018; Oesch, 2017). The tracking not only influences further skills development (Angelone and Ramseier, 2012; Baumert et al., 2006; Tomasik et al., 2018) but also further education trajectories (Gomensoro and Meyer, 2021; OECD, 2002; Scharenberg et al., 2016, 2017). Although track allocation claims to be achievement-based, allocation criteria strongly vary within and between cantons, are lacking valid discriminatory power and are most of all highly socially selective even while controlling for academic achievement (Felouzis et al., 2011; Kronig, 2007; Neuenschwander et al., 2012).

In Switzerland, compulsory schooling ends after lower-secondary level. With the exception of two among the 26 Swiss cantons (Geneva and Ticino), students are neither legally entitled nor obliged to pursue their education at upper-secondary level (and further; for more detail see www.edk.ch/en/education-system).

A second important setting of the course occurs at the end of compulsory schooling: Upper-secondary level education in Switzerland includes, on the one hand, general education programmes geared toward later attendance of university studies (approx. 30% of a school leavers' cohort). However, the large majority of Swiss compulsory school leavers (over 60%) attends vocational education and training (VET). There are VET programmes in over 200 training professions with varying academic requirements, their duration ranging from 2 to 4 years. Most programmes are provided in a “dual” form, that is, learners attend a (usually larger) part of their training “on the job” in a training company, while the remaining time is spent in (vocational) school. VET trainees in this type of programme sign an apprenticeship contract with the training firm and receive a (modest) salary. The Swiss dual VET system relies heavily on private firms, which are free to train apprentices or not. Only about a third of Swiss employers offer VET training (Aepli et al., 2021). Upon completion of their training, as of 2004, VET trainees in Switzerland obtain three types of upper-secondary level diploma which provide access to the labor market and/or further training at post-secondary and tertiary levels: the federal certificate (2 years of training), the federal diploma (three to 4 years of training) and the vocational baccalaureate^8^.

It should also be noted that the Swiss labor market is strongly segmented and stratified by the occupation-specific formal credentials obtained through VET. The occupation-specific segment of the labor market, by far the largest in Switzerland, is subdivided in itself into some hundred occupation-specific sub-segments, each of which is based on an occupation-specific VET diploma. Access to jobs in these sub-segments is generally restricted to VET graduates holding the respective diploma (Sacchi et al., 2016). While this mechanism is known to smooth transitions from VET to the labor market, it is also known to substantially hamper labor market mobility between occupations (see e.g., Buchs et al., 2015; Mueller and Schweri, 2015).

It is further important to mention that in Switzerland, there is a strong linkage between the tracks that students attend at lower-secondary level education and the programmes of upper-secondary education. This pertains not only to the access to general education programmes, which is largely restricted to students having attended tracks with high or extended academic requirements. De facto (if not formal) restrictions also apply to the access to VET programmes. As a general rule, students having attended lower-secondary tracks with low academic requirements have substantially reduced opportunities to access training professions with high occupational status, irrespective of their academic achievement (BFS/TREE, 2003; Gomensoro and Meyer, 2021; Meyer and Sacchi, 2020).

At tertiary level, Swiss students enroll in either universities, universities of applied sciences (UAS), universities of teacher training or programmes for professional education and training (PET; for more detail, see www.edk.ch/en/education-system/diagram). While tertiary completion rates are fairly high among general education graduates from upper-secondary level (over three quarters), they are, by international standards, low among VET graduates (at approximately one third only; see Meyer, 2016 for more detail).

With regard to overall educational attainment, roughly 90% of youth complete upper-secondary education.^9^ At tertiary level, women's completion rate has risen to 40 per cent by the beginning of the 2020s, compared to 30 per cent among men. Post-compulsory education trajectories are strongly genderized: At upper-secondary level, more than one third of young women enroll in academic tracks, while this share is at one fourth among young men. Contrariwise, almost two thirds of young men are to be found in VET programmes, compared to only half of all young women. Furthermore, VET training professions are also markedly gendered: Among the 20 most frequently chosen training professions, more than half are extremely male- or female-dominated (with a share of more than 90 percent of one sex).^10^

With particular respect to educational expansion and its role in the O-E-D triangle, it can be held that in a historical perspective, expansion in Switzerland took place relatively late and haltingly by international standards, especially at tertiary level education. Mostly due to the VET system expanding in the early post-war decades, upper-secondary graduation rates among the male population rose to approximately two thirds until 1970, while women lagged considerably behind (approx. 40%).^11^ Traditionally, Swiss education policy has strongly restricted the share of youth enrolling in general education programmes at upper-secondary level that are geared to provide access to university studies (below 25%). The policy rationale behind this restriction has always been (and still is) that the academic tracks are not supposed to “weaken” the supply of well-achieving students for the VET tracks (Leemann, 2025; Meyer, 2018; SCCRE, 2014). Hence and largely due to the fact that transition from upper-secondary VET to higher education became more common and widespread only after 2000,^12^ tertiary level educational attainment has remained under 10 per cent of the adult population way into the 1970s and only reached 20 percent in the late 1990s. Moreover, the share of foreign students enrolled in Swiss universities has always been high, which has traditionally led to an overestimation of higher education graduation rates of the autochthone population.

### 3.2 (Transition to the) labor market

To date, the Swiss economy has enjoyed a period of virtually uninterrupted favorable macro-conditions throughout more than two decades. By international comparison, Switzerland has been less economically affected by disruptive macro-events such as the financial crisis of 2008/9, the Covid pandemic and the recent upheavals of the war in Ukraine. While economic growth has somewhat slowed in recent years, unemployment and inflation levels remain constantly low, while employment rates and standards of living are among the highest among OECD member states (OECD, 2024b). With regard to the labor market, there has been a long-lasting shortage of skilled, high-qualified labor (Adecco and SMM, 2023). As a consequence and ever since Switzerland's adhesion to the Agreement on the Free Movement of Persons (AFMP) with the European Union in 2002, annual net labor immigration (mostly) from EU countries has been ranging from 40.000 to over 80.000 individuals annually, i.e., the equivalent of one half to an entire Swiss birth cohort.^13^

Generally, the macro-economic context described above is also reflected in the school-to-work transition and labor market integration of young Swiss adults. Labor market prospects are relatively favorable even for young workers who leave the education system with low- to medium-level qualifications and credentials. However, tertiary-level graduates enjoy exceptionally high employment rates and considerable income advantages over those who lack tertiary-level credentials (Gomensoro et al., 2017).

As already briefly outlined in Section 3.1, the linkage between the “market” of/for VET trainees and the general labor market in Switzerland is strong. In many respects, VET trainees thus de facto enter the labor market when they take up their training. In the past, the VET-labor market linkage has led to considerable volatility as regards the availability of VET training places, particularly during and after the recession of the 1990s, which led to a substantial shortage of (VET) training opportunities.

### 3.3 Relevant changes of the macro-context

In the 16 years that lie between the launch of the two TREE cohorts, the macro-context in which educational pathways and transitions are embedded has witnessed substantial changes, including reforms of the education system which can be expected to affect youths' educational trajectories. In the essentially federalist Swiss education system(s), this involved, e.g., the unification of the previously very heterogeneous cantonal programmes and curricula within primary and lower-secondary level education.^14^ In some cantons, this has resulted in a later tracking^15^ of students at lower-secondary level of education, which is widely held to curb social selectivity (see, e.g., Bauer and Riphahn, 2006).

Between 2000 and 2016, a number of cantons have furthermore enacted or implemented reforms of lower-secondary education geared to mitigate tracking separation and thus improve student mobility between tracks. While some cantons introduced or strengthened cooperative or integrative programmes during the period in question, others in turn tended to even reinforce their separative models.^16^ Overall, it remains inconclusive whether tracking has increased, remained stable or decreased between 2000 and 2016. (Moser 2008) claims that in any case the differences between the cantonal models tend to be only gradual, as even the models labeled as cooperative or integrated incorporate elements of tracking.

At upper-secondary level, the amendment of the Vocational and Professional Education and Training Act (VPETA), effective as of 2004, fell into the period between the launch of the two TREE cohorts. Among other things, the reform integrated occupational fields into federal VET legislation that were previously regulated at the cantonal or industry level (particularly in the health and agriculture sectors). Another major amendment was the introduction of the federal VET certificate, a 2-year VET programme designed to grant an upper-secondary degree for low-achieving students.

As to changes regarding VET training supply, the first TREE cohort completed compulsory education at the end of a period marked by a recession during the 1990s. Owing to the linkage between labor market and the supply of VET training places, this resulted in a pronounced lack of the latter (Hupka-Brunner et al., 2011). Cohort comparisons indicate a moderate easing of this discrepancy between demand and supply for the second TREE cohort. However, the match between demand and supply remains difficult due to the volatility of local and/or occupation-specific VET “markets.”

At upper-secondary level, the period between the launch of the two TREE cohorts is marked by a distinct increase of learners enrolled in general/academic programmes (from approximately one quarter to approximately one third of an age cohort) at the expense of VET trainees, whose share has decreased from approximately two thirds to below 60 percent.^17^ This shift translates to a decrease of the share of youth who are (exclusively) gainfully employed in their early twenties, as the large majority of graduates from the general/academic programmes enroll in university studies at tertiary level education.

## 4 Research questions and hypotheses

Against the background outlined so far, the overall research question of this paper is whether and to what extent intergenerational mobility patterns have changed among Swiss cohorts that have recently made their transition from education to employment: In the case of the first TREE cohort (average birth year 1984), the transition is complete, in the case of the second TREE cohort (average birth year 2000) it has already progressed substantially.

Owing to the availability of the presently released data, progress of longitudinal observation for the second TREE cohort—and thus for comparisons across cohorts—is restricted to 5 years between the end of compulsory school (9th grade, average age of 15–16) and average age 20 to 21, at which point the majority of the cohort has concluded upper-secondary education. We assume that at this point the “destination” within the O-E-D triangle is far from having been reached, particularly among the 40%−45% share of the cohort that has not yet concluded upper-secondary education and/or can be expected to pass on to tertiary-level education. Owing to the fact that close to 60% of a Swiss school-leavers' cohort enroll in VET programmes at upper-secondary level, we are nevertheless able to determine the occupational status of the training profession that they have opted for (see Data & Methods Section below for more detail). As occupational mobility and propensity to take up tertiary-level studies is known to be low in the low- to medium-level strata of the Swiss occupational system (Kriesi et al., 2022), we can therefore expect this measure (i.e., occupational status of training profession) to be fairly predictive of the “final destination.”

In choosing this approach, we make a conscious decision of focus: Contrary to most analyses that examine intergenerational (im)mobility, our focus is not on higher education attainment, but on the stratification within and between the professions that VET trains for, i.e., the low- to medium strata of the occupational system.

Against the overarching research interest of this paper, i.e., to what extent parental status is predictive of children's status with regard to professions for which VET training and credentials are prerequisite, we formulate the following research questions (RQ) and related hypotheses (H):

RQ1: Do we observe changes in the relationship between parental status (origin) and status attainment (destination) between the two observed TREE cohorts?
H1: As the macro-conditions of VET training opportunities were more favorable for the second cohort than for the first and in view of the educational reforms implemented between 2000 and 2016, we expect the effect of parental status on children's status to be weaker for cohort 2 than for cohort 1.
RQ2: Has the effect of tracking at lower-secondary level education on status attainment changed across cohorts?
H2: As the macro-conditions of VET training opportunities were more favorable for the second cohort than for the first and in view of the educational reforms implemented between 2000 and 2016, we expect the effect of lower-secondary tracking to be weaker for cohort 2 than for cohort 1.
RQ3: To what extent does tracking at lower-secondary level mediate intergenerational status transmission? Has the extent of direct effects and indirect effects (i.e., mediated by tracking at lower-secondary level) of parental status changed across cohorts?
H3: In view of the particularly unfavorable context conditions of the first TREE cohort with regard to VET training opportunities and given the reforms implemented between the two cohorts (see Section 3), we expect the indirect effect of parental status through tracking on children's status to be weaker for cohort 2 than for cohort 1.

## 5 Data and method

The Swiss TREE study provides data for two (compulsory) school-leaving cohorts that are representative at the national level: TREE1 (initial *n* = 6,343; TREE, 2019) completed compulsory schooling in 2000, TREE2 (initial *n* = 8,429; TREE, 2024) reached the end of compulsory schooling in 2016, i.e., at a time when said reforms had taken effect. Both cohorts were followed up at annual intervals throughout the first 6 years of their post-compulsory trajectories. Presently, the released data cover an observation span of 5 years for both cohorts. They provide an excellent opportunity to run detailed and comprehensive comparative analyses of the two cohorts, taking into account a wide range of individual, familial and institutional/contextual characteristics. The TREE data of both cohorts provide detailed information on respondents' educational and employment pathways as well as on educational attainment and socioeconomic status of their parents. TREE's multi-cohort design thus allows to examine whether the mentioned institutional reforms and societal changes have brought about a change with regard to the influence of parental social background (origin) on educational trajectories and attainment on the one hand and on occupational status attainment on the other (destination).

In order to study the relation between parental and children's positions, we rely on the linear hierarchical scale of occupations' earning potential (OEP) recently developed by (Oesch et al. 2025). The OEP scale measures the median earning of each occupation and expresses it in percentiles of the earning distribution of the entire workforce. That means that OEP values for each occupation are between 0 and 100 percentiles and that, for instance, occupations that have a value of 50 are in the middle of the earning structure. The scale was constructed on annual earnings data of full-time employed men and women that cover several decades for Germany, Sweden, Switzerland, the UK and US. The authors validate the scale by testing its association with education and intergenerational social mobility and by comparing it with the international socio-economic index (ISEI; see Ganzeboom et al., 1992). They conclude that the two scales (OEP and ISEI) yield closely comparable results. However, OEP has the advantage of simplicity, ease of interpretation and parsimony. Unlike scales such as ISEI that are based on several dimensions (e.g., education, earnings, and prestige) it is based on a single dimension (earning potentials), and the results are easy to interpret) (Oesch et al., 2025). In addition, OEP proved to be relevant across several decades and European countries, making it particularly suited for intergenerational comparison in the Swiss context.

The OEP values are operationalized in the TREE data as following. Fathers' and Mothers' occupations are reported by the TREE respondents in the baseline survey of each cohort (in 9^th^ grade). We recode it from ISCO88 (first cohort) and ISCO08 (second cohort) 4-digit level into ISCO08 3-digit level by relying on the *iscogen* module in Stata (Jann, 2019). We then transform it into OEP percentiles according to (Oesch et al. 2025) and retain the highest parental OEP.

With regard to the TREE respondents themselves, we construct the OEP they have attained 5 years after completion of compulsory schooling (i.e., at around age 21).^18^ At this time, most respondents have completed upper-secondary education and started either tertiary level education or taken up gainful employment. Given the variability of situations in which young adults find themselves (education, employment, internships, unemployment, etc.), we define OEP according to various relevant criteria and depending on available ISCO^19^ information.

First, we translate ISCO codes related to vocational education, employment and internships into OEP and retain the highest value for each respondent. We do so assuming that it is highly probable that they reach at least the occupational status of the profession in which they do their training or internship.^20^ However, in most of the cases, they are not in two situations (education, employment or internship) simultaneously. In the datasets, we have ISCO information for respondents that are exclusively economically active (98% of them), in vocational education and training at upper secondary or tertiary level (99%), and in internships (76%).

Second, in the event that we do not have ISCO information from the previously mentioned criteria (mainly when respondents are not in employment, education or training or in rare cases of missing ISCO information related to the previously mentioned situations) we consider the ISCO and respective OEP related to vocational degree obtained in previous years. In doing so, we are able to include 56% of the respondents who are not in employment, education or training. Finally, we do not have the information needed to calculate an OEP for respondents undertaking general upper secondary or general tertiary education (universities), as these programmes do not prepare for a specific occupation (no ISCO information) and because student jobs cannot be considered to be predictive of their future occupation. Out of a sample of respondents from panel wave 5 of 4,506 for the first cohort and of 4,501 for the second, we have OEP values for 2,831 and 2,964 respondents respectively. After exclusion of some cases that have missing values in in relevant variables used in the analysis, we end up with samples of 2,674 valid cases for the first cohort and of 2,820 for the second. The data available at the time of publication of this study, which extends up to the average age of 21, allows us to study the intergenerational transfer of the status of a specific part of the two cohorts, i.e., mainly those who engaged in VET programmes.

In order to assess the consequences of these sample restrictions on sample skewness and results, we have duplicated our analyses by drawing on the full sample of the first cohort and measuring OEP at average age 30, i.e., when most respondents have completed their formal education and find themselves at various stages of their early professional careers. The results of this assessment corroborate the skewness of the available sample in the main analysis at average age 21 (see Appendix). On average, OEP of the subsample undertaking general upper secondary and university education is considerably higher for both respondents and their parents. Moreover, average OEP of the sample used in our analysis continues to increase between average ages 21 and 30, as respondents engage in further training and advance in their professional career.

Returning to our main analysis, we proceed as follows in the ensuing results section: In a first descriptive analysis step (see Section 6.1), we present OEP averages of both respondents and their parents and compare them across cohorts. In a further step (see Section 6.2), we perform linear regressions of both cohorts separately to examine the extent to which parental OEP affects children's OEP. In the regression analysis (see part 6.2.), we first run model 1 that includes respondents' OEP at around age 21 (dependent variable) and parental OEP (independent variable), and we add lower-secondary tracking in model 2. The comparison of the cohort-specific coefficients allows us to examine RQ1 (coefficients of parental OEP) and RQ2 (coefficients of lower secondary tracking). We then run model 3 that includes the control variables and model 4 that includes both control variables and lower-secondary tracking, as well as a robustness checks. Control variables include gender, migration status and language region, which are known to be strongly associated with education and status attainment (OEP). To test if differences are significant across cohorts, we rely on the z-test developed by (Clogg et al. 1995) and assessed by (Paternoster et al. 1998). This test provides results that are similar to the estimation of a single model for both cohorts that adds interaction terms between cohort and the tested variables.^21^ Finally (see Section 6.3), we evaluate to what extent the effect of parental OEP on respondents' OEP occurs directly or indirectly through the attendance of specific lower-secondary tracks (mediator as defined in RQ3) in model 5 and model 6 (including control variables). Causal mediation models are performed with the structural equation model estimation command (*sem*). All analyses are performed with Stata18 (StataCorp, 2023), account for TREE's complex sample design and are weighted in order to compensate for panel attrition (Sacchi, 2011, 2024).

## 6 Results

### 6.1 Descriptive statistics

With regard to the descriptives of our analysis, Table 1 shows that the average Occupational Earning Potential at around age 21 is very similar for both cohorts, at around 41 on the scale. For both cohorts, OEP averages are substantially below 50, the median of the OEP distribution. This can be explained by the fact that we do not have any way to assign an accurate OEP value to those who undertake general education at upper-secondary or tertiary level and will reach later in their life course higher levels on the OEP scale. Furthermore, we are observing OEP at a point in time when a non-negligible share of the sample under study is still in training and/or will further progress on their educational and professional trajectories.^22^

**Table 1:** Descriptive statistics.

	**Mean and standard error in brackets, or %**	
**Cohorts**	**Cohort 1**	**Cohort 2**
**Dependent variable**		
OEP at average age 21	40.92 (0.58)	40.81 (0.53)
**Independent variable**		
Parental OEP (highest)	49.08 (0.69)	53.90 (0.68)
**Mediator variable**		
Track attended at lower secondary level		
Basic/low requirements	35.75%	35.88%
Extended requirements	45.98%	48.20%
High requirements	18.27%	15.91%
**Control variables**		
Sex		
Female	45.78%	46.66%
Male	54.22%	53.34%
Migration status		
No migration	76.96%	71.92%
Second generation migrant	10.09%	20.96%
First generation migrant	12.95%	7.12%
Language region		
German	76.69%	76.16%
French	20.25%	20.45%
Italian	3.06%	3.39%
Sample size	2,674	2,820
(Weighted results)		

Average parental OEP is higher than that of their children in both cohorts, at 49 for the first and almost 54 for the second, indicating that in their early twenties, respondents have not yet reached their parents' OEP level. This difference is more marked for the second cohort, where parents' average OEP level is higher than in the first. The difference in parental OEP between the cohorts is statistically significant. It most likely reflects changes in social stratification in Switzerland, marked by a decreasing share of the working class and an increase of the middle class (Oesch, 2023). Between 2000 and 2016, (Oesch 2023) also observes an increase in the population's level of education and an increase of highly qualified immigration [also observed by (Wanner and Steiner 2018)].

### 6.2 Regression analysis

We now turn to the regression models to assess the effect of parental OEP on their children's OEP and to identify any differences between the two cohorts (RQ1). We begin by comparing the regression coefficients of the models without covariates (see model 1 in Tables 2, 3). These “empty” models allow us to estimate the overall OEP transmission effects observed in the data. Parental OEP has a significant effect on children's OEP, and to a comparable extent for both cohorts. The coefficient for the second cohort is slightly higher, but there is no statistically significant difference between cohorts. If we increase parental OEP by one percentile, children's OEP rises by 0.11 (cohort 1) and 0.13 (cohort 2). We also observe that the proportion of variance explained (see R-squared value of model 1 in Tables 2, 3) is slightly higher for the second cohort than for the first, which suggests a slightly stronger link between parents' and children's OEP for the second cohort than for the first. In sum, we observe, at a stable level across cohorts, a very strong stratification mechanism at play with respect to the low- to medium-level occupational positions under study here, thus contradicting our first hypothesis (H1).

**Table 2:** Linear regression on respondents' OEP at average age 21. Cohort 1.

**Models**	**Model 1**			**Model 2**			**Model 3**			**Model 4**		
	**Coefficient**	**Sig**.	**SE**	**Coefficient**	**Sig**.	**SE**	**Coefficient**	**Sig**.	**SE**	**Coefficient**	**Sig**.	**SE**
Parental OEP (highest)	0.1075	^***^	0.0269	0.0762	^**^	0.0265	0.1026	^***^	0.0268	0.0728	^**^	0.0258
Lower-secondary track attended (ref. Extended requirements)												
Basic/low requirements				−5.5543	^***^	1.1348				−6.3961	^***^	1.1282
High requirements				0.5547		1.2972				1.3413		1.2295
Sex (ref. Female)												
Male							7.8943	^***^	0.8795	8.1951	^***^	0.8553
Migration status (ref. no migration status)												
Second generation migrant							1.5045		1.7456	2.4441		1.6796
First generation migrant							−1.4480		1.3474	0.2834		1.3833
Language region (ref. German)												
French							−1.3069		1.0572	−2.6379	^*^	1.0420
Italian							0.3416		2.3024	2.0627		2.2611
Constant	35.6448	^***^	1.3782	39.0655	^***^	1.5546	31.8919	^***^	1.5808	35.1318	^***^	1.6793
R-squared	0.0167			0.0445			0.0795			0.1163		
Sample size	2,674			2,674			2,674			2,674		

OEP scale ranges from 1 to 100; Sig.: Levels of significance ^***^ *p* < 0.001, ^**^ *p* < 0.01, ^*^ *p* < 0.05, ^+^ *p* < 0.01; SE: standard error; weighted results.

**Table 3:** Linear regression on respondents' OEP at average age 21. Cohort 2.

**Models**	**Model 1**			**Model 2**			**Model 3**			**Model 4**		
	**Coefficient**	**Sig**.	**SE**	**Coefficient**	**Sig**.	**SE**	**Coefficient**	**Sig**.	**SE**	**Coefficient**	**Sig**.	**SE**
Parental OEP (highest)	0.1297	^***^	0.0251	0.1122	^***^	0.0263	0.1236	^***^	0.0239	0.1060	^***^	0.0250
Track attended at lower-secondary level (ref. Extended requirements)												
Basic/low requirements				−3.8185	^***^	1.1485				−4.7303	^***^	1.1356
High requirements				−1.5580		1.5533				−1.2819		1.4593
Sex (ref. Female)												
Male							7.8629	^***^	1.0459	8.1679	^***^	1.0534
Migration status (ref. No migration status)												
Second generation							1.0745		1.4406	1.9389		1.4371
First generation							−3.6645	+	2.0138	−2.3174		1.9517
Language region (ref. German)												
French							−1.7343		1.1303	−2.2217	+	1.1925
Italian							1.0274		1.8403	1.6471		1.8892
Constant	33.8146	^***^	1.4124	36.3783	^***^	1.6753	30.3048	^***^	1.4409	32.7941	^***^	1.6433
R-squared	0.0247			0.0352			0.0871			0.1023		
Sample size	2,820			2,820			2,820			2,820		

OEP scale ranges from 1 to 100; Sig.: Levels of significance ^***^ *p* < 0.001, ^**^ *p* < 0.01, ^*^ *p* < 0.05, ^+^ *p* < 0.01; SE: standard error; weighted results.

When we add the school track attended at lower-secondary level (see model 2 in Tables 2, 3), we see that the effect of parental OEP persists at a high level for both cohorts. However, the coefficient related to parental OEP decreases to a larger extent for the first cohort than for the second. Attending a track with basic/low academic requirements compared with one with extended requirements reduces OEP by 5.5 percentiles in the first cohort and by 3.8 percentiles in the second (there is no difference between those in high with those in extended requirement streaming). This confirms that attending the basic/low requirement tracks strongly hampers access to vocational training with high OEP and, subsequently, to higher-paying occupations. When we include lower-secondary tracking in the model, the proportion of variance explained also increases more strongly for the first cohort than for the second. This suggests a larger effect on respondents' OEP and stronger mediation by the type of lower-secondary track attended with respect to the linkage between parental OEP (origin) and respondent OEP (destination) for the first cohort (compared to the second). We can thus confirm the second hypothesis (H2) which expects that the effect of tracking at lower-secondary level on later OEP (destination) has decreased across cohorts (RQ2).

We also estimated two regression models including gender, migration status and language region as control variables (see model 3 without and model 4 with the school requirement at lower secondary level in Tables 2, 3). The first observation we can make is that for both cohorts, the addition of these three control variables does not affect the coefficients relating to the parental OEP. In model 4 (see Tables 2, 3), the negative effect of attending the basic/low stream is confirmed and even slightly reinforced when the control variables are added. We also observe a very large effect of gender on OEP. In both cohorts, men, on average, have an OEP that is approximately 8 percentiles higher than that of women. This gender gap is also observed later in life, at around age 30, for the first cohort and when considering also those who undertook general education at secondary and tertiary level (see Appendix). Additional analyses (not published) show that at age 30, the OEP gender gap has even widened to 11 percentile points for those who attended VET programmes at upper-secondary level (i.e., essentially the subsample under study in our main analysis here). Contrariwise, we observe no statistically significant gender gap among those who attended general education programmes. Our results strongly suggest that the Swiss VET system incorporates a substantial OEP penalty for women, which they are not only unable to catch up on, but which even increases in the course of their twenties. Migration status does not appear to have any effect (*p* < 5%) on OEP at this level of aggregation (first and second generations). We observe a negative effect of being a migrant on OEP for the second cohort, but this effect is only statistically significant in model 3 and at the < 10% level. Finally, with regard to language region, we observe a negative effect for the French-speaking part of the country both for the first and, albeit to a lesser extent (*p* < 10%), the second cohort. This is likely to reflect regional differences with respect to the societal appreciation of VET (vs. general education),^23^ but also the to the labor market and the supply of VET training places.

### 6.3 Mediation of the effect of parental OEP through lower-secondary tracking

As shown above, the impact of parental OEP is similar for both cohorts (RQ1), and the effect of tracking for the second cohort is smaller than for the first (RQ2). We are now turning to RQ3 and H3, i.e., the question whether lower secondary tracking mediates the association between parental (origin) and children's (destination) OEP. We carry out these analyses for models which include the variable lower-secondary tracking without (model 5) and with additional control variables (model 6) of Table 4 for the first cohort and of Table 5 for the second.

**Table 4:** Mediation of the effect of parental OEP on respondents' OEP through lower secondary tracking at average age 21. Cohort 1.

**Models**		**Model 5**				**Model 6**			
		**Coefficient**	**Sig**.	**SE**	**%**	**Coefficient**	**Sig**.	**SE**	**%**
Parental OEP (highest)	Direct effect	0.0784	^**^	0.0254	73	0.0764	^**^	0.0247	70
	Indirect effect	0.0291	^***^	0.0063	27	0.0331	^***^	0.0065	30
	Total effect	0.1075	^***^	0.0253	100	0.1096	^***^	0.0250	100
Tracking at lower-secondary level		3.4352	^***^	0.6390		3.9123	^***^	0.6160	
Sex						8.1691	^***^	0.9376	
Migration status						0.2954		0.6792	
Language region						−1.2753	+	0.7373	
Significance test of indirect effect	Delta *p*-value		^***^				^***^		
	Sobel *p*-value		^***^				^***^		
	Monte Carlo *p*-value		^***^				^***^		
Significance test of mediation effect	Baron and Kenny	Partial mediation				Partial mediation			
	Zhao, Lynch and Chen	Partial mediation				Partial mediation			

OEP scale ranges from 1 to 100; Sig.: Levels of significance ^***^ *p* < 0.001, ^**^ *p* < 0.01, ^*^ *p* < 0.05, ^+^ *p* < 0.01; SE: standard error; weighted results.

**Table 5:** Mediation of the effect of parental OEP on respondents' OEP through lower secondary tracking at average age 21. Cohort 2.

**Models**		**Model 5**				**Model 6**			
		**Coefficient**	**Sig**.	**SE**	**%**	**Coefficient**	**Sig**.	**SE**	**%**
Parental OEP (highest)	Direct effect	0.1193	^***^	0.0262	92	0.1054	^***^	0.0261	89
	Indirect effect	0.0104	+	0.0057	8	0.0134	^*^	0.0059	11
	Total effect	0.1297	^***^	0.0245	100	0.1188	^***^	0.0245	100
School requirement at lower secondary level		1.6329	^*^	0.8040		2.1050	^**^	0.7963	
Sex						8.0817	^***^	1.0264	
Migration status						−0.3526		0.9032	
Language region						−1.3285		0.8352	
Significance test of indirect effect	Delta *p*-value		+				+		
	Sobel *p*-value		+				+		
	Monte Carlo *p*-value		+				+		
Significance test of mediation effect	Baron and Kenny	Partial mediation				Partial mediation			
	Zhao, Lynch and Chen	Partial mediation				Partial mediation			

OEP scale ranges from 1 to 100; Sig.: Levels of significance ^***^ *p* < 0.001, ^**^ *p* < 0.01, ^*^ *p* < 0.05, ^+^ *p* < 0.01; SE: standard error; weighted results.

For the first cohort, we observe that the indirect effect of parental OEP via lower secondary tracking on their children's OEP amounts to 27% (model 5) and 30% (model 6 with control variables) and is statistically significant (*p* < 1‰) in both models. Partial mediation is confirmed by the three significance tests of indirect effect (*P* < 1‰) as well as by the Baron & Kenny and Zhao, Lynch & Chen significance test of mediation effect (partial mediation). The indirect effect is significantly lower for the second cohort (8% for model 5 and 11% for model 6), whereas the total effect of parental OEP was similar to that of the first cohort. For the second cohort, the indirect effect is not confirmed by the tests for model 5 and not strongly confirmed for model 6 (given a statistical significance level of *P* < 10%).

These differences suggest important changes in the role played by lower-secondary tracking *per se* and as a mediator of occupational parental origin and children's destination.

Concerning the effect *per se*, we notice that the coefficient relating to tracking is twice as high for the first cohort as for the second 3.4 and 1.6 (respectively in Tables 4, 5). As regards the mediation effect, one hypothesis is that the influence of social origin characteristics on the selection processes at play in the transition from primary to lower-secondary education is less marked in the second cohort than in the first. However, as the overall transmission effect remains stable across cohorts, we assume that status mobility has not increased over time and that parental origin is influencing children's destination via other mechanisms (represented in this analysis by the direct effect). Moreover, the additional analysis of the first cohort (see Appendix) suggests that between average age 21 and 30, the effect of social origin increases while the mediation effect of lower-secondary tracking on occupational stratification remains stable. This is true irrespective of whether we account for the entire first cohort sample (i.e., including general education trajectories) or the VET subsample under study in our main analysis here.

## 7 Conclusions

In the wake of post-World War II educational expansion and economic boom of the “*trente glorieuses*,” the decreasing extent of intergenerational status transmission and increasing social mobility suggested a societal shift toward more equal opportunity irrespective of social origin. However, more recent debates draw an inconclusive picture of how social mobility has evolved since the mid-1970s (see Section 2).

In the tradition of the Origin-Education-Destination (O-E-D) paradigm initially proposed by (Blau and Duncan 1967), empirical evidence in the present debate tends to be flawed by two major shortcomings: Firstly, it often relies on “historical” and/or retrospective data, thus reflecting O-E-D mechanisms that lie largely in the past. Secondly, it often draws on cross-sectional data, thus leaving us in the dark as to intermediate steps and processes that lie between “origin” and “destination.”

This is where the present analysis sets in. Drawing on longitudinal data of two Swiss school-leavers' cohorts that have left compulsory school relatively recently (2000 and 2016 respectively) we examine intergenerational status transmission within the O-E-D triangle in its early stages, i.e., between age 15–16 (i.e., at the end of compulsory school) and early adulthood (approximately age 21).

While it is obvious that the process of status attainment is far from being “concluded” at the end of this stage, we make a virtue out of the necessity related to the restricted observation span of the younger cohort. What comes to our help is the Swiss education system's particularity that approximately two thirds of the country's youth attend VET programmes at upper-secondary level. Although many VET graduates continue their educational trajectories beyond their upper-secondary degree and age 21, their training profession, due to the strong linkage between VET and labor market position, is highly predictive of the occupational status that they will attain in their early career (irrespective of the fact whether they are already gainfully employed or still in training at age 21). Furthermore, more than half of all apprentices who graduate have already entered the job market by the age of 21.

Our analysis design attempts to model this two-step path dependency for the two cohorts' subsamples that attend VET programmes at upper-secondary level by including the school track attended at lower-secondary level in our analyses, which plays a decisive role in determining which VET programmes are accessible to young people. In contrast to many studies focussing on intergenerational status transmission of academic tracks, our focus thus rests on a population that can be expected to attain low- to medium-level positions in the hierarchy of the Swiss occupational structure and on the central research question whether we are able to observe any changes in status attainment within the O-E-D triangle.

In sum and contrary to our expectations (see Section 4), the present study provides compelling empirical evidence of the extent to which social origin impacts on status attainment among two recent school-leavers' cohorts in Switzerland. Despite major educational reforms (see Section 3) and improvements with regard to the supply of VET training places at upper-secondary level, we observe no changes of social (im)mobility patterns across cohorts. In line with our hypotheses, the impact of lower-secondary tracking has decreased across cohorts. This suggests that institutional changes of the education system do seem to play a (mediating) role with regard to institutional path-dependencies. However, this has not led to a reduction of overall intergenerational status transmission, at least not among the low- to medium-level occupations under study in this contribution. The persistence of (the overall extent) of this transmission despite the decreasing impact of lower-secondary tracking across cohorts leaves room for various interpretations. On the on hand, it tends to support the inequality persistence paradigm (see Section 2), which assumes that more advantaged families are prone to find their way to counteract “equalizing” measures within the education system, thus securing the “headstart” of their offspring. On the other hand, we may assume that the educational reforms implemented between 2000 and 2016 (outlined in Section 3) have proved to be insufficient to attenuate social reproduction patterns. This would be in line with criticisms vis-à-vis Swiss educational policy that point out the discrepancy between formal and *de facto* permeability within the strongly stratified education system. The critical positions hold that the latter will remain marginal as long as the former is not reinforced by measures that directly foster educational equity (i.e., reduce or eliminate structural barriers and allocate more resources and support to disadvantaged students; see, e.g., Oesch, 2017; Maag Merki, 2016).

A further striking finding of our analysis is the strong gender effect. If we introduce gender as a control variable in our models, we observe an average effect of no < 8 percentile points to the disadvantage of females. This reflects and confirms the marked genderisation of the Swiss VET system and labor market, in which “female-dominated” professions systematically lead to substantially lower occupational status (and vice versa; see, e.g., Hupka-Brunner and Meyer, 2023).

From a methodological perspective, our analysis highlights the advantages of multi-cohort and longitudinal data in capturing long-term dynamics of intergenerational status transmission. Furthermore, relying on Occupational Earning Potential (OEP; Oesch et al., 2025) as a new analytical tool for measuring (occupational) status allows us to account for individuals who are still engaged in vocational education and training, rather than having to restrict our analysis to those who have already made the transition to the labor market. The OEP measure also allows us to study changes in social hierarchy over time (see the additional analysis for the first cohort at age 30 in the Appendix).

Our analysis is not without limitations. Firstly, it is important to acknowledge that respondents, as already stated above, are for from having reached their highest occupational earning potential at average age 21. The peak of occupational status typically is attained between age 35 and 50 (Shahbazian and Bihagen, 2022; Bihagen et al., 2024). This limitation underlines the need for continued analysis to assess whether the observed mobility patterns are persistent, weakened or reinforced as cohort careers unfold over time. Our additional analyses at age 30 (see Appendix) highlight the respective dynamics at play throughout the third decade of the first cohort's trajectories.

A second limitation relates to the limited occupational information available for individuals engaged in general education trajectories, including university graduates, who have not yet entered the labor market at this stage in their lives, for whom we cannot attribute an OEP. This limitation hampers a full capture of cohort mobility patterns, particularly among those from high-OEP family backgrounds. Our additional analyses in the Appendix suggest a substantial sample bias toward individuals with low parental OEP, which may result in underestimating the role played by social origin and lower-secondary tracking.

A third limitation relates to the difficulty of disentangling the effects of educational reforms on the one hand and the supply of VET training places on the other. Our findings suggest that improvements in the supply of VET training places (which are at least partly linked to the fluctuations on the labor market) across cohorts may have contributed to the decreasing effect of lower-secondary tracking. However, further research is needed to disentangle the relative impact of educational reforms and economic or other macro-structural changes.

A final limitation of our analysis is due to the fact that it does not account for covariates at the local, cantonal or regional levels. In a markedly federalist and heterogeneous education system such as Switzerland's, variability of cantonal or regional education regimes and supply of different programmes, regional labor markets (companies that are willing to hire VET trainees) and economic cycles can be expected to also affect O-E-D mechanisms and are not fully captured by the design of the present analysis.

## Data Availability

Publicly available datasets were analyzed in this study. This data can be found here: TREE (2024). Transitions from Education to Employment, Cohort 2 (TREE2), Panel waves 0-5 (2016-2021) (Version 3.0) [Dataset]. SwissUbase, FORS. https://doi.org/10.48573/69q6-b447; TREE (2019). Transitions from Education to Employment, Cohort 1 (TREE1), Panel waves 0-9 (2000-2014) (Version 5.0) [Dataset]. SwissUbase, FORS. https://doi.org/10.23662/FORS-DS-816-7.

## Footnotes

^2^O = Origin, E = Education, D = Destination (i.e., attained [occupational] status)

^3^European Social Survey.

^4^Programme for the International Assessment of Adult Competencies.

^5^(Bernardi et al. 2019, p. 4) life course cube essentially captures three dimensions or axes of life course trajectories: time, life domains and level of social aggregation (from individual to societal).

^6^Contrary to international standards, the “official” numbering of (compulsory) years of education in Switzerland includes two years of kindergarten. Officially, Swiss students hence leave compulsory school after eleven years of schooling (for details, see https://www.edk.ch/en/education-system/diagram).

^7^See Indicators of Education regularly updated by the Swiss Federal Statistical Office (FSO); (in German and French only).

^8^The vocational baccalaureate (VC) is a supplementary degree at upper-secondary level that is conferred upon completion of the federal VET diploma. It is either obtained parallelly to the latter or consecutively after one to two years of further study/training. The VC grants access to the Universities of Applied Sciences (UAS).

^9^See Indicators of Education, regularly updated by the Swiss Federal Statistical Office (FSO); (in German and French only).

^10^See Indicators of Education regularly updated by the Swiss Federal Statistical Office (FSO); (in German only).

^11^All educational attainment figures of this paragraph draw on Swiss Federal Statistical Office figures of Table T 40.02.15.08.11, retrieved from the SFO website on 20 February 2025.

^12^In the 1990s, Switzerland introduced a VET-based equivalent to the academic baccalaureate, the vocational baccalaureate, which grants access to the then newly implemented Universities of Applied Sciences (UAS). By the mid-2010s, completion rates of the vocational baccalaureate had risen to approximately 15 percent (and stayed at that level since). Approximately 60 percent (i.e., around 10% of an age cohort) of vocational baccalaureate holders undertake UAS studies.

^13^For details, see the annual reports of the Swiss government's AFMP observatory (www.seco.admin.ch).

^14^See the Intercantonal Agreement on Harmonisation of Compulsory Education (HarmoS).

^15^In a number of cantons, transition to the (tracked) lower-secondary level took place after 4^th^ or 5^th^ grade of primary. Harmonization resulted in a uniform transition after 6^th^ grade for all cantons (except Ticino).

^16^The degree of integration/cooperation vs. separation essentially hinges on the number of lessons during which students of the different tracks are taught within one and the same class/classroom. In some separative tracking models, students of the various tracks do not share classes/classrooms at all (and in some cases attend school on separate premises). In the most cooperative/integrative models, track-specific students share all the lessons in the same class/classroom but follow track specific curricula in the subjects that are relevant for track allocation (usually mathematics, teaching language and first foreign language). Less than 3 percent of all students in Switzerland attend lower-secondary education without any tracking whatsoever (see SFO Indicators of education).

^17^See www.tree.unibe.ch/results.

^18^Although status attainment is not ‘completed' at this stage, we assume that this point of observation is already meaningful for various reasons: in Switzerland, about two-thirds of the country's young people attend VET programmes at upper-secondary level. Due to the strong link between VET and labor market position, this is a solid predictor of the occupational status that they will achieve in their early careers (regardless of whether they are already employed or still in training at the age of 21). Furthermore, more than half of all VET graduates have made the transition to the labor market by the age of 21.

^19^International Standard Classification of Occupations.

^20^18 months after graduation, three quarters of Swiss VET graduates are working in the occupation that they have been trained in. Five years after graduation, this share is at 50% (BFS, 2020).

^21^As a robustness-check, we also run a single regression model with interaction terms between cohorts and tested variables. Results showed no statistically significant difference between the cohorts and in the interaction terms.

^22^See the Appendix in Section 14 that documents the substantial OEP increase between average ages 21 and 30 for the cohort 1 sample under study in this paper.

^23^In the French-speaking part of Switzerland, the share of students enrolled in VET programmes at upper-secondary level is substantially lower than in the German-speaking part, while general education is more highly valued.
