# Supplementary material for: Intergenerational status transfer and post-compulsory pathways in a changing education system: a comparison of two Swiss school-leavers' cohorts
Source: Front Sociol. 2025 Sep 1;10:1585464. doi: 10.3389/fsoc.2025.1585464 (PMC12434961; doi:10.3389/fsoc.2025.1585464)
Supplement: Supplementary file 1 [file Table_1.pdf]

frontiers

Gomensoro A, Hupka-Brunner S and Meyer T (2025) Intergenerational status transfer and post-compulsory pathways in a changing education system: a comparison of two Swiss school-leavers' cohorts. *Front. Sociol.* 10:1585464. doi: 10.3389/fsoc.2025.1585464

1 Appendix: OEP regression analysis for the full sample of the first TREE cohort at age 30

In this appendix, we replicate our analyses for the first cohort but take into account the observation period that the published data actually cover, i.e., up to average age 30.[1] This provides us with a more complete picture of the parents to children occupational status transmission for the entire representative cohort of compulsory school leavers in 2000 as, at age 30, when the large majority of the cohort, including those who have undertaken tertiary level studies, has made the transition to the labour market. We replicate the descriptives (see Article Section 6.1), the regression (Section 6.2) and mediation (Section 6.3) analyses with the OEP observed at average age 30 and compare them with the OEP observed at average age 21. This allows us to assess the skewness of the available sample at age 21 (referred in the following paragraphs as the subsample included in the main analysis) and its impact on the main analysis of this paper. The replication also allows us to assess the OEP among the part of the sample for which we have no OEP data at average age 21 (referred as the general education subsample, i.e. those undertaking general upper secondary and university education).

As to the descriptives, we observe that the OEP increases from an average value of 40.9 (see Article Table 1 in Section 6.1) to an average value of 48.7 at average age 30. Thus, observing the OEP too early is not ideal, as OEP increases sharply during this career period. This clearly suggests that status attainment is far from having reached its peak at age 21, the reference age we have chosen in view of the limited observation period of the second cohort. The descriptive statistics also confirm that the subsample available for the main analysis largely excludes the upper social strata. The general education subsample of the cohort has a mean OEP of 55.7 and a mean parental OEP of 56.3 (compared with 48.7 and 49.1 respectively for the subsample included in the main analysis). It should also be noted that at average age 30, both the subsamples have reached the average OEP of their parents.

With regard to lower-secondary tracking we see that the subsample that we drew for the main analysis is substantially overrepresented in having attend a track with low requirements (36%) and conversely underrepresented among the track with high requirements (18%). Contrariwise the opposite is true for the general education subsample (14% and 50% respectively).

With regard to the regression analysis (see Appendix Table 1), we observe that the effect of parental OEP at age 30 is twice the size of the effect at average age 21 (see Appendix Table 2 in the main text). This is first and foremost due to the fact that the model now includes individuals who have attended university studies and hence attain a higher professional status.

---

[1] We have conducted a further panel wave at average age 35, but the data are not published yet.

Additional analyses (not included in this paper), show that the general education subsample is characterized by a stronger association between parents' and respondents' OEP than the sample considered in the main analysis (mainly VET students). In addition, the later measurement of OEP includes the contributions of tertiary education, as well as career development towards better-paid positions. As far as the effects of tracking are concerned, the negative effect of attending a track with low requirements is confirmed and, if we draw on the entire cohort sample, even accentuated over time. It seems that those who have attended the low track enrol in upper-secondary programmes that provide limited opportunities for further studies or professional mobility up to age 30. Only 3% of this group enrol in university studies by age 23, while this share amounts to 30% among those who have attended a track with high academic requirements (Meyer and Bertschy 2011).

As to the control variables, the effect of gender on OEP remains striking at later stages of the trajectories. However, additional analyses (not published in this paper) show that among the general education subsample, gender-related effects of OEP are only about one third (significance at the $p<10\%$ level) of the size observed in the subsample drawn on for the main analysis (significance at the $p<1‰$ level).

With regard to the role of lower-secondary tracking as a mediator for intergenerational OEP transmission, the comparison of Appendix Table 2 with Table 4 in the main body of the paper shows that tracking mediates the effect of parental OEP on respondents' OEP to a similar extent (around 26-27%; significance at the $p<10\%$ level).

To sum up, we observe that the skewness of the sample drawn on in the main analysis excludes mainly cases with high parental and respondents' OEP on which we have no available to estimate an OEP. Our main analysis thus tends to underestimate the extent of intergenerational status transmission in the entire cohort sample. In addition, we notice that OEP differences by gender are more marked in VET-based occupations than in occupations taken up after general education. Finally, the mediation effect of lower-secondary tracking is pervasive for the entire first cohort, most likely due to the strong path dependencies at play throughout the education system.

Appendix Table 1 Linear regression on respondents' OEP at average age 30. Cohort 1.

Models	Model 1			Model 2			Model 3			Model 4		
	Coefficient	Sig.	SE	Coefficient	Sig.	SE	Coefficient	Sig.	SE	Coefficient	Sig.	SE
Parental occupational earning potential (highest)	0.2026	***	0.0403	0.1381	***	0.0404	0.1765	***	0.0405	0.1143	**	0.0399
Track attended at lower-secondary level (ref. Extended)												
Basic/low requirements				-9.2620	***	2.0339				-10.0569	***	2.1160
High requirements				2.0445		1.8778				3.5421	+	1.9825
Sex (ref. Female)												
Male							7.6623	***	1.6490	8.8721	***	1.5914
Migration status (ref. No migration status)												
Second generation							1.2181		2.3042	2.3377		2.4153
First generation							-4.9074	+	2.5447	-1.3849		2.6254
Language region (ref. German)												
French							0.2128		1.8754	-2.8767		2.0225
Italian							-1.0304		3.4291	1.1404		3.1502
Constant	42.6788	***	2.2823	48.1250	***	2.3307	40.5719	***	2.7645	45.1831	***	2.6618
R-squared	0.0409			0.0889			0.0824			0.1398		
Sample size	2,781			2,781			2,781			2,781		

OEP scale ranges from 1 to 100; Sig.: Levels of significance *** p<.001, ** p<.01, * p<.05, + p<.01; SE: standard error; weighted results.

Appendix Table 2 Mediation of the effect of parental OEP on respondents' OEP through lower secondary tracking at average age 30. Cohort 1.

Models		Model 5				Model 6			
		Coefficient	Sig.	SE	%	Coefficient	Sig.	SE	%
Parental occupational earning potential (highest)	Direct effect	0.1508	***	0.0446	74	0.1257	**	0.0425	68
	Indirect effect	0.0518	***	0.0136	26	0.0597	***	0.0142	32
	Total effect	0.2026	***	0.0444	100	0.1853	***	0.0429	100
Track attended at lower-secondary level		5.5702	***	1.1043		6.4137	***	1.0947	
Sex						8.9679	***	1.6403	
Migration status						-0.6189		1.3324	
Language region						-1.2570		1.3940	
Significance test of indirect effect	Delta p-value	+				+			
	Sobel p-value	+				+			
	Monte Carlo p- value	+				+			
Significance test of mediation effect	Baron & Kenny	Partial mediation				Partial mediation			
	Zhao, Lynch & Chen	Partial mediation				Partial mediation			

OEP scale ranges from 1 to 100; Sig.: Levels of significance *** p<.001, ** p<.01, * p<.05, + p<.01; SE: standard error; weighted results.

2 References

Meyer, Thomas, and Kathrin Bertschy. 2011. 'The Long and Winding Road from Education to Labour Market: The TREE Cohort Six Years After Leaving Compulsory School'. In *Transitionsprozesse Im Jugendalter: Ergebnisse Der Schweizer Längsschnittstudie TREE*, edited by Manfred Max Bergman, Sandra Hupka-Brunner, Anita Keller, Thomas Meyer, and Barbara E. Stalder, 92–119. Zürich: Seismo. https://doi.org/10.33058/seismo.30881.
